# A Preliminary Stability Assessment of Three State-of-the-Art CAD/CAM Materials Under Human Gingival Cell Culture

**DOI:** 10.3390/polym17020221

**Published:** 2025-01-17

**Authors:** Eduard Gatin, Stefan Iordache, Ana Maria Iordache, Alexandra Totan (Ripsvki), Antoniu Moldovan, Catalin Luculescu

**Affiliations:** 1 Faculty of Medicine, University of Medicine ‘‘Carol Davila’’, 050474 Bucharest, Romania; 2 Faculty of Physics, University of Bucharest, Atomistilor 405, 077125 Magurele, Romania; 3 Optospintronics Department, National Institute for Research and Development in Optoelectronics—INOE 2000, 077125 Magurele, Romania; stefan.iordache@inoe.ro (S.I.); ana.iordache@inoe.ro (A.M.I.); 4 Faculty of Dentistry, University of Medicine “Carol Davila”, 050474 Bucharest, Romania; alexandra.totan@umfcd.ro; 5 Plasma and Radiation Physics, National Institute for Laser, 077125 Magurele, Romania

**Keywords:** CAD/CAM materials, Vicker’s hardness test, Raman spectroscopy, cell culture, surface modifications, structural integrity

## Abstract

CAM/CAD composites are widely used as dental restoration materials due to their resistivity to wear. The purpose of this study was to determine the effect of human gingival fibroblast cells on three different computer-aided design/computer-aided manufacturing (CAD/CAM) hybrid materials with resin-based composites (RBC) and to assess their stability following cell growth. The CAM/CAD dental materials were investigated in different conditions as follows: (i) cells (human gingival fibroblasts, HFIB-Gs) incubated over the material for each sample, denoted as A; (ii) reference, the raw material, denoted as B; and (iii) materials incubated in DMEM medium, denoted as C. We employed Vicker’s hardness test, EDS, SEM, and AFM measurements as well as Raman spectroscopy to carefully characterize the surface modifications and the structural integrity of the CAM/CAD materials before and after fibroblast cell culture. The analysis of the surface in terms of morphology, roughness, structure, and plastic deformation presented no significant difference after incubation in cells or in media, proving their extraordinary stability and resilience to biofilm formation.

## 1. Introduction

In the last few years, new advances in computer-aided design/computer-aided manufacturing (CAD/CAM) techniques have reached a dominant role in restorative dentistry [1,2,3]. The boost was based on advances in adhesive techniques and the introduction of new materials [4]. The CAD/CAM technique is offering great advantages, including an improved standardization of the restoration manufacturing process and low production costs [5,6]. CAD/CAM techniques allow restorations to be fabricated in a one single visit to the dentist, maybe the most important achievement of the method [7]. The focus of this study was composite resins specifically designed for CAD/CAM milling and not for layered modeling, such as 3D printing. The materials that are used for CAD/CAM restorations can be classified into ceramics and composites [8]. While ceramics are the preferred material for indirect restorations in clinical practice [9], exhibiting a low fracture toughness and high brittleness, the others have the big advantage of less enamel wear on the antagonist tooth [10].

The scientific and technological development of dental materials grows exponentially; every year, new materials are introduced on the market with the aim of giving dentists new clinical alternatives for the treatment of high-risk patients [11]. Thus, all new materials are focused on the application of new technologies such as CAD/CAM systems [12,13]. Hybrid ceramics seek to combine the optical and mechanical properties of ceramics and composite resins, two of the most used materials in restorative dentistry [14].

Fractures are common in CAD/CAM restorations, regardless of whether they are made of ceramics or composites [15]. When faced with such failures, specialists have the choice of replacing the failed restoration or opting for a complete repair. Total replacement is not always the best option in many clinical situations because it can potentially harm healthy tooth tissue and is more time-consuming. Repairing the failed restoration has the advantage of minimizing time and reducing the risk of trauma to surrounding tissues [16,17,18].

Ceramics and glasses are often used as bone substitutes because of their chemical compatibility and/or similar mineral composition with bones. However, in clinical applications, there are some difficulties related to the brittleness and low reliability of the mechanical behavior of ceramics [19]. Therefore, it is meaningful to search for ceramics with good mechanical properties that also possess bioactive properties. ZrO_2_-based materials have excellent properties because of their toughness and resistance, better than other bio ceramic materials. Along with their good biocompatibility, zirconia materials find many applications in bone surgery and dental applications. Eutectic ceramics are arousing considerable interest due to their good mechanical behavior, but they should be enhanced by reducing aging sensitivity and improving osteointegration performance [20]. Although they exhibit excellent mechanical and optical properties as well as biocompatibility, ceramic materials are fragile, rigid, and hard to repair. On the other hand, composites are easy to manipulate and repair, more flexible, and less abrasive on the antagonist tooth, but their poor wear resistance and difficulty in obtaining good polish put them at a disadvantage compared to ceramics [14]. Conventional ceramics produce highly esthetic restorations; however, some studies have identified a higher incidence of failure of these materials, possibly caused by their rigidness and abrasive effect on the antagonist tooth [20].

The main advantages of resin materials are clear: color modulation and stability that mimic natural enamel and high flexural resiliency that ensures adequate mechanical properties [21,22]. Furthermore, because they are commercialized as high-density suspensions, they are versatile and can be used to fabricate all types of prosthetics, and they are better suited to repair damaged intraoral composites and enhance patient comfort [23]. In real-life situations, these properties are affected primarily by the specific material microstructure and by the environmental conditions in the mouth. Because of the softer, low-hardness polymeric phase that creates a weak interphase bond, the CAM/CAM materials are subjected to lower durability and structural failure, which is translated to a shorter life span [24]. This comes in contradiction with a recent study [25] surveying the longevity of CAM/CAD materials used in implant manufacturing, which calculated a mean annual failure rate of 0.74%, for 12 years (for 5 years, the failure rate was 3.3%). Decementation and fracture of the ceramic were the main failure types, and no failure of the composite was observed within a 2.5-year period.

Another existing research gap refers to the multitude of commercial products that exist on the market today, which require a detailed analysis and a comprehensive characterization in order to address the differences between existing commercial materials. To fill this gap, we design a study that evaluates the stability in the incipient stage (the first 48 h) of three classes of CAM/CAD materials used regularly in all dental offices. Thus, following our previous studies [26,27], this paper evaluates the morphostructural changes that affect the surface of three different, state-of-the-art CAD/CAM materials following human gingival epithelial cell culture. Two previous studies [28,29] showcased the influence of CAM/CAD materials on the development, apoptosis, and terminal differentiation of the fibroblasts, but they do not give any information on the state of the surface of the composite materials. To the best of our knowledge, this is the first study that focuses entirely on the characterization of the surface of the CAM/CAD materials following the first 48 h of incubation of human gingival fibroblasts. The CAM/CAD materials we investigated are representative of a particular class of fillers: glass–fiber-based (Trinia) composites, zirconia-based composites (Coritec ZrO), and feldspar enriched with Al_2_O_3_ (Vita Enamic). We aim to assess the stability of the selected materials in terms of morphology, roughness, structure, and plastic deformation. Since most studies investigate the influence of microstructure on the optical properties [30] or the attachment methods [31] or evaluate the degradation of the materials in endogenous media (cariogenic biofilm, salivary enzymes, staining solutions, and gastric acid) [32,33,34], we investigated (1) the structural integrity of the composite by Raman spectroscopy and (2) the morphology and roughness of the interface following cell culture through SEM/EDX and AFM following the incipient development stage. Vicker’s hardness test was employed to assess the flexural strength of the materials before and after cell culture.

## 2. Materials and Methods

For our study, three suitable materials were selected for CAD/CAM techniques that have a dominant role into the restorative dentistry in Romania. The three materials belonged to one of the three classes of fillers: (i) glass fibers, (ii) ZrO_2_ based, and (iii) feldspar based. The aim of the investigation was to evaluate and to compare the selected materials from the physicochemical and mechanical point of view and to assess the difference in term of stability between classes following incubation of human gingival fibroblasts. The complete characteristics of the materials involved in our study are presented in Table 1.

All samples from each material were cut from commercial blocks (they were attached to a sample holder and sectioned into smaller pieces with a cutting machine) and shaped as discus for easy handling, with diameter of d = 13 mm and thickness of h = 3 mm (A_sample_ = 3.87 cm^2^). The surface was roughly polished to a normal smooth surface with silica carbide paper (no 400, no 600, and no 1200). Three samples were fabricated in this way for each of the composites.

The above-mentioned dental materials were investigated in different conditions as follows and labeled by letter: (i) A—cells (human gingival fibroblasts, HFIB-Gs) incubated over the material for each sample; (ii) B—reference, the rough material; (iii) C—materials incubated in DMEM (Dulbecco’s modified eagle’s medium, Gibco Invitrogen, Darmstadt, Germany).

### 2.1. Cell Culture

Human gingival fibroblasts (HFIBs) were purchased from Innoprot (Derio, Spain). The cells were supplied in vials with ≈500.000 cryopreserved viable cells [35,36]. The HFIB cells were seeded according to standardized protocols [37] for adherent cultures using DMEM with 150 µL poly-L-lysine 1 mg/mL in 10 mL sterile water and incubated at 37 °C until a confluency of 90% was obtained.

Cells grew onto CAD/CAM materials: The three CAM/CAD materials were coated in collagen type I from rat tail (1 mg/mL supplied as sterile liquid in 0.001% acetic acid, Collagen I-Cell Culture Surface Coating Kit from Innoprot, Derio, Spain), and the HFIBs were allowed to grow for 48 h. Following proliferation of the cells, the three materials were washed with saline solution and 70% ethanol solution and investigated to assess their stability.

We chose HFIB-Gs over other components of the oral environment because they promote connective tissue regeneration and are responsible for creating a strong, well-connected biofilm on surfaces. Adult human gingival fibroblasts (HGFs), the most abundant cells in the oral cavity, are essential for maintaining oral homeostasis. Also, compared with other tissues, adult oral mucosal wounds heal completely without scarring. HGFs have shown immense capabilities in the regeneration of both hard and soft dental and periodontal tissues, which highlights their potential as a valuable tool in regenerative dentistry. HGFs have been used as seed cells in a sandwich-tissue-engineered construct, and due to the significant role of HGFs in osteogenesis and mineralization, periodontal defects were completely repaired in dogs [38].

### 2.2. Raman Spectroscopy

Investigation was performed using a NRS-7200 microspectrometer (JASCO, Tokyo, Japan) (λ = 532 nm, output power *p* = 5.5 mW, and spectral resolution at least 4 cm^−1^), in the Raman shift range 300–3500 cm^−1^ for all investigated samples. The integration time was 30 s with 20 accumulations. Raman signal was collected in backscattering geometry perpendicular on the sample surface through a 10× magnification objective LMPFLN 10x (Olympus, Tokyo, Japan). The laser spot diameter on sample was about 20 microns in diameter. Raman spectra were multipoint wavelength calibrated using a pure polypropylene sample. Experimental data were recorded in the same geometrical conditions in three different points in order to avoid local heating of the samples. Selected values for Raman peaks intensities were obtained after normalization (to the unit, average values) applied to raw data. Differences in peak intensities on raw spectra reflected the differences in the quantities of the chemical components for investigated specimens. Sensitive qualitative/quantitative information may be obtained according to the Raman spectra shape (including the fluorescence information) using raw data without smoothing [39,40].

### 2.3. Energy Dispersive X-Ray Spectroscopy (EDS) and Scanning Electron Microscopy (SEM)

The equipment employed in our study was a SEM microscope FEI Inspect S, equipped with a secondary electron detector in low vacuum and a solid state BSE detector, plus an auxiliary micro analytic EDS—Si(Li) radiation detector (EDAX Sapphire UTW, 130 eV resolution). The EDS measurements provide accuracies in 0.1–1% range, being suitable for measurements of major and minor components but not for trace analysis.

### 2.4. Atomic Force Microscopy

AFM micrographs were collected with a XE100 AFM (Park Systems, Suwon, Republic of Korea) in non-contact mode, using commercial probes OMCL-AC160TS (Olympus Europa, Hamburg, Germany). Topography maps were recorded for each sample on at least two different locations, on 40 μm × 40 μm areas. In the 3D-rendered topography images, the horizontal and vertical scales have the same magnification. The color palette highlights the local height as well as the slope of the surfaces. The RMS (Root Mean Square) roughness was calculated for each scanned area.

### 2.5. Hardness Test

We employed Vicker’s test (Future-Tech Corp., Model FM-700 and Serial number XM0190, Kawasaki, Japan) since it is easier to use than other hardness tests. The advantage of this method is that the required calculations are independent of the size of the indenter and are specific to the materials investigated.

## 3. Results

### 3.1. Raman Spectra

**Figure 1:**
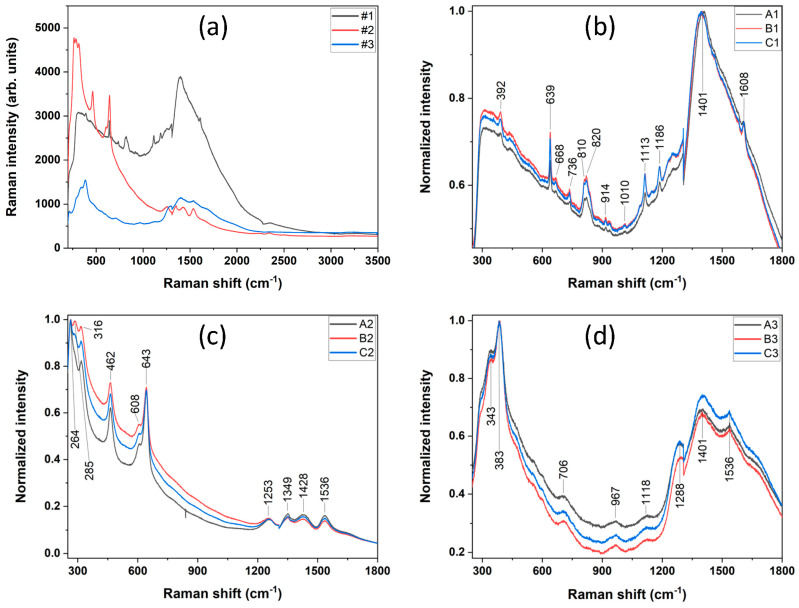
(**a**) Raman spectra for all 3 materials involved in this study in 200–3500 cm^−1^ range; (**b**–**d**) Raman spectra for materials in all 3 conditions: #1 Trinia (**b**); #2 Coritec Zr (**c**); #3 Vita Enamic (**d**).

The Raman spectra in the 300–3500 cm^−1^ range for the three investigated materials in the initial conditions are shown in Figure 1.

The assignment of the most important Raman peaks is presented in Table 2. The only overlap of the peaks for composites #1 and #2 is 639 cm^−1^ (#1)/643 cm^−1^ (#2), which corresponds to different compounds: SiO_2_, for the Trinia, respectively, and ZrO_2_ for the Coritec. For #3 and #2, the overlap is present at 1288 cm^−1^ (#3)/1253 cm^−1^ (#2).

### 3.2. EDS (Energy-Dispersive X-Ray Spectroscopy)

**Figure 2:**
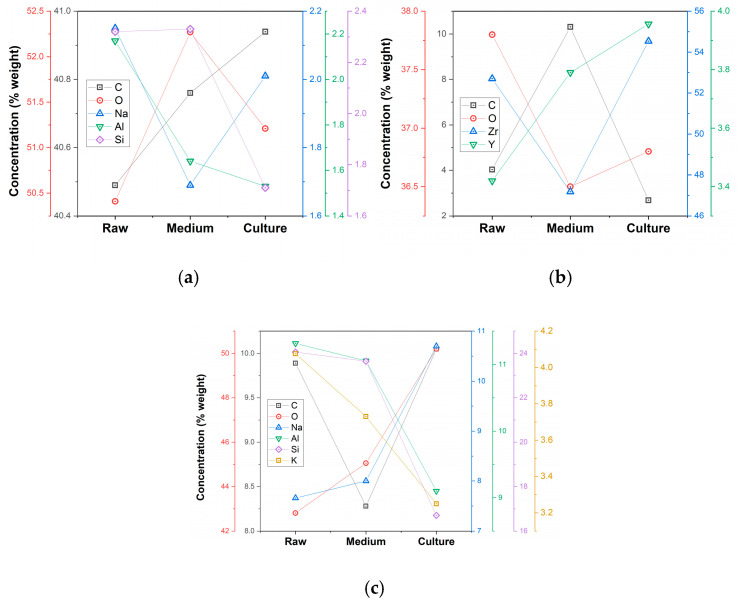
Energy-dispersive X-ray spectroscopy results for major and minor components for samples #1 (**a**), #2 (**b**), and #3 (**c**) under all studied conditions: raw samples, cell medium affected, and cell culture affected.

The chemical composition of the three samples is presented in Figure 2 and Tables S1–S3 in Supplementary Files for major and minor elements. For the base materials (B), there is a close agreement with the manufacturers’ specifications.

### 3.3. Scanning Electron Microscopy (SEM)

**Figure 3:**
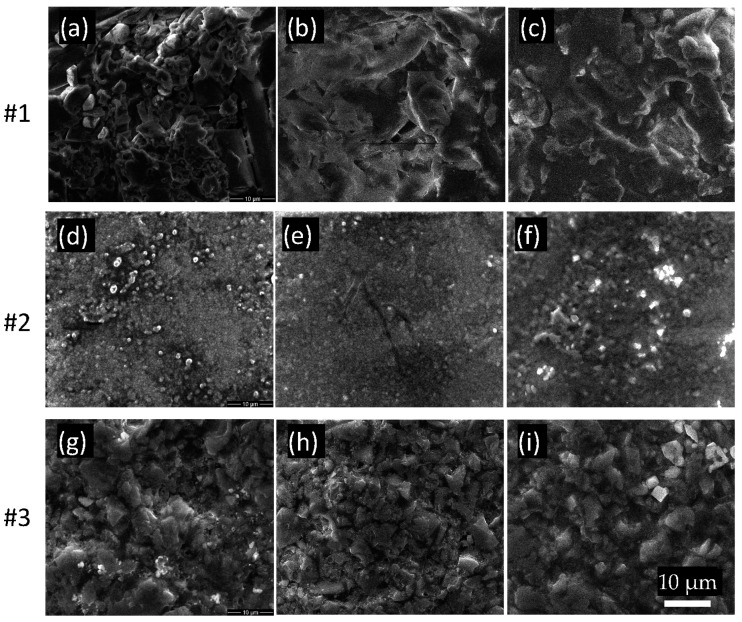
Sample #1 (**a**–**c**), #2 (**d**–**f**), and #3 (**g**–**i**), micrographs under different treatments: (**a**,**d**,**g**) as produced; (**b**,**e**,**h**) DMEM incubated samples; (**c**,**f**,**i**) after HFIB cell culture. The magnification is 5000×, and the 10 µm scale is presented in (**i**).

For this investigation, SEM was performed for all samples with magnification ×5000 (Figure 3).

### 3.4. AFM

**Figure 4:**
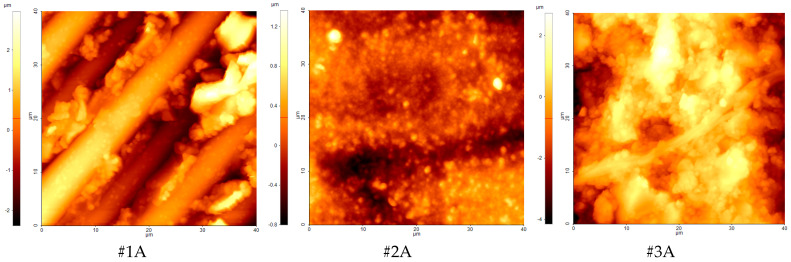
Random AFM images for the surface of #1A, #2A, #3A samples of CAM/CAD materials following the incubation of the HFIB-Gs on their surface (40 × 40 µm).

The results of the SEM are sustained by the AFM measurements. The AFM images for the three CAM/CAD materials following the incubation of the HFIB-G are presented in Figure 4 (all the images for the reference and medium incubation are shown in the supplementary file, Figure S1). The AFM topography was performed on 2–3 randomly chosen areas on each sample, and the images were obtained in tapping mode. The topography of the samples exhibits a non-uniform surface. Sample #1A shows the glass fibers embedded into the epoxy resin (Trinia). There are no modification on the surface of the sample following the incubation of HFIB-Gs. The same results are obtained for the other two samples, Coritec ZrO and Vita Enamel, which show insignificant surface modifications following the biofilm growth.

**Figure 5:**
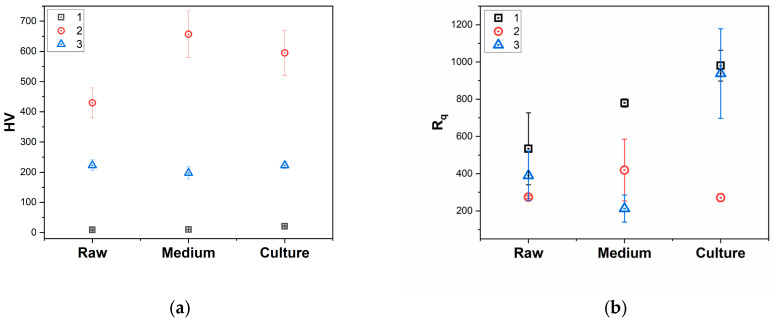
(**a**) Vicker’s microhardness results for the three samples; (**b**) Rq values for the samples resulted from AFM measurements.

The RMS roughness values were calculated from AFM topography images and are presented in Figure 5b as Rq. The topography of the samples is not homogeneous; it varies strongly from one point to another, even for the same sample. It is most likely inherent to the typical processing steps involved in sample preparation.

### 3.5. Microhardness

Measurements for Vickers’ microhardness are relevant for the characterization of polymer materials. The mechanical properties [47] are strongly correlated with microhardness. Figure 5 and Table S5 (Supplementary Files) shows the results of three individual measurements per sample, the average value of the results, and the standard deviation (SD).

## 4. Discussion

We analyzed the first material (#1), which is composed of 55% glass fiber and 45% epoxy matrix; this half-organic, half-inorganic composition is revealed in the Raman spectrum by two broad peaks: one below 600 cm^−1^ corresponding to the inorganic phase and one ≈ 1500 cm^−1^ corresponding to the organic phase. The second material (#2) is composed mainly of yttria tetragonal zirconia polycrystals, showing a Raman-active zone below 1000 cm^−1^. The third compound (#3) has a Raman behavior similar to the first composite, with two Raman-active regions below 600 cm^−1^ and ≈1500 cm^−1^.

Following cell culture on the surface of the composites and incubation in DMEM medium, the Raman spectra showed little structural modifications compared with the initial, rough material. As can be seen from the overlay of the Raman spectra for each material (Figure 1b–d), the ceramics are extremely stable, the variations in peak intensity from one reference to another being negligible. The Raman spectra also revealed that Coritec has a mixture of tetragonal (>80%) and cubic ZrO_2_ (<20%), which corresponds to what other authors described.

The analysis of Raman spectra indicated a high portion of organic matter corresponding to the epoxy resin matrix for samples #1 and #3, which is consistent with the finds in our previous works [39,40]. Raman spectroscopy comes as a complementary method to FTIR for the characterization of dental ceramics [48] and bioglass [49]. The Raman spectrum for the initial materials is overlaid in Figure 1a and can be corroborated with the composition of each material. The main finding is that all the investigated composites are Raman active below 2400 cm^−1^. An important observation is the fact that sample #2 shows few organic bonds, and the Raman spectra reveals the high inorganic character expressed by the producer in the product sheet. The surface modifications of the samples were further investigated by EDS means, which sustained the Raman results. For the samples incubated in DMEM, there were minimal changes detected. Thus, for sample #1 (Trinia, a mixture of glass fiber + epoxy resin), the elements of C and N corresponding to the epoxy resin recorded a decrease in the wt%, while O and Na showed an increase. This behavior could be due to a good adhesion of the DMEM medium on the surface of the materials once chemical elements such as N (from L-Glutamine) and Na (from sodium pyruvate and sodium bicarbonate) are identified.

Regarding the organic part for samples #1 and #3, the list of chemical elements is richer than manufacturer specification. For sample #2, some traces of organic matter (maybe impurities) are noticed. Other elements such as Al, Zr, Cl, Ca, K, Fe, and Ni presented an increase in the wt% following DMEM incubation, which is possibly due to contamination. The same trend is observed for sample #3 (Vita, feldspar ceramic with epoxy resin), with elements such as K, Na, and Cl showing up as traces possibly from the cleaning solution (saline solution was used for cleaning the materials before measurements).

In comparison, the EDS for the materials incubated in HFIB-Gs show an interesting behavior: for sample #2, Zr and Y show an increase (measured as wt%) than the standard material. At the same time, N and O show an abrupt decrease (nitrogen is reduced to 0). This unusual behavior could be explained by the degradation of the surface of the material due to cell development. The cells adhere to the surface of the material and begin biodegradation of the material. This, in turn, is revealed in the EDS as an increase in the wt% of the elements that compose the material (Zr and Y increase, while O decreases which indicates that the ZrO_2_ and Y_2_O_3_ are broken-down, with O_2_ being consumed by the cells) [50,51]. For the increase of C, possible explanations could be either incomplete washing of the materials or the destruction of polymeric chains following degradation of the material by cells. For sample #1, an increase in the majority of elements is observed: the most evident is the three times increase of C (probably from the organic material that was incompletely washed). Other increases occur for N, O, Zn, Al, Si, Cl, Ca, and Na, with P, Fe, and Ni in trace amounts (a possible contamination). Sample #3 is the most stable one from the three, with an almost unmodified composition across the incubation materials (HFIB-Gs and DMEM). For this sample, only the levels of O show a slight decrease, while the Cl, Y, and Na show a small increase. Since Y is not present in the original (standard) sample, this can only be present due to a contamination, while Na and Cl are remnants from the washing process.

Further analysis of the surfaces of the materials were performed by SEM and AFM means. Our previous studies [52,53] on dental restoration materials have shown that SEM microscopy is a powerful tool to reveal the minuscule changes in the morphology of the surface. The results show that for sample #1, after the incubation of the HFIB-Gs and after the washing of the cells, the organic material is not optically visible, and it is not adherent to the polymer. The lamellar structure of the fiber glass is only in small proportion covered by organic matter that is responsible for the increases in C and O shown in the EDS spectrum. For the DMEM, sample #2 shows little adhesion to the surface or corrosion (Figure 3e).

For sample #2, the organic material is less adherent to the beaded zirconia crystal structure, and the organic contamination is less evident. However, the surface of the inorganic material after the HFIB-G incubation shows a corroded area, with valleys and hills, which indicate that the cells will “eat away” the components needed for development and will ignore the ones they do not need. This is in full accordance with the results of the EDS measurements. For the DMEM incubation, we do not observe any corrosion of the sample. Comparing the surfaces of sample #1 and sample #2, we observe that sample #1 promotes the formation of films (Figure 3b), which are difficult to wash away, while in the case of sample #2, this biofilm does not form (Figure 3e).

For sample #3, the organic material resulting from the HFIB-G incubation is even less adherent to the resin, with no evidence in SEM images and with small increases shown by EDS measurements. The clear polymer resin structure shown in Figure 3h,i is conserved after both HFIB-G and DMEM incubation states. The SEM images corroborate the EDS measurements that sample #3 best conserves the original surface morphology in oral media. AFM measurements could not indicate any substantial modification of the surface due to a high intrinsic roughness of the surface of the samples. This is due to the short incubation period for the gingival cells selected for this study since three studies on different brushing and grinding mechanisms to evaluate the biofilm formation and material stability showed that the brushing affects the gloss and surface roughness values [32,47,54]. However, biofilm formation is connected to the type of material of the sample: resin-based materials have lower adhesion of the biofilm, while ceramic- or resin-based materials have higher biofilm adhesion. The possible influences of the studied exposures to cells or the medium might be “hidden” by this high roughness topography (typical RMS roughness values of hundreds of nm, up to more than 1 μm).

Since Vicker’s test was the only available tool, we could not perform the flexural strength of the sample after cell culture. Focusing on sample #1, the values for microhardness are similar for the sample that underwent cell incubation compared to the standard (B) and DMEM samples (C). By corroborating these results with the SEM, AFM, and EDS results, one can conclude that the film formation on the surface of the sample does not influence the strength of the material. The roughness of the samples are varying slightly following cell culture (Figure 5b).

For sample #2, both HFIB-G cell culture and DMEM incubation induce an increase in Vickers’ microhardness compared to the standard sample. This increase is explained by the fact that the deposition of organic material (without forming a biofilm, as shown by the SEM and EDS measurements) induces the formation of adhesions between the organic and inorganic matter, thus strengthening the structure of the composite.

For sample #3, the values for the microhardness are similar in all investigated conditions, this material being the most stable. This material does not promote cell proliferation or biofilm formation. Compared with PMMA (polymethylmethacrylate) and PLA (polylactic acid), which are susceptible to biodegradation via increased moisture absorption [55], the polymers contained by sample #3 (urethane dimethacrylate and triethylene glycol dimethacrylate) form a compact structure with small pores, where moisture cannot diffuse. Studies investigating the influence of water uptake on the resistance of dental cements and mechanical resistance of dental epoxy resins have shown that the dental materials are exhibiting dimensional changes exceeding the calculated values [56]. This is due to the hybrid nature (organic–inorganic) of the dental materials: although the inorganic part has a higher elasticity module, the organic part is less rigid and tends to concentrate the stress in a particular point (the one with the least resistance) [57,58,59]. However, in our case, elasticity is not of particular interest since the scope of the paper is to investigate the stability of our samples in HFIB-G culture and DMEM incubation.

The results are consistent with the few reports [28,29] that indirectly deal with the stability of the CAM/CAD materials incubated in human gingival fibroblasts. The materials show a high physico-chemical stability under cell growth, and although organic matter adhesion (cells or medium) is sometimes seen, it does not influence the stability and resilience of the composite. Sample #3 (Vita Enamic) is particularly stable, our results being validated by Jennes et al. [28]. Our results show that UDMA is not corroded by gingival cells, although Yu et al. [60] concluded that this polymer is corroded by *S. mutans* following a 14-day culture. Alamoush et al. [29] showed that #3 has the lowest cytotoxicity and the least cell proliferation for HGIF-Gs.

**Strengths:** To the best of our knowledge, HGIF-Gs were not tested with #2 (Coritec ZrO) or #1 (Trinia), our results coming forward to fill this gap. This is particularly true for Raman spectroscopy for these materials, for which the attribution of bands was difficult to perform. Difficulties were also encountered at AFM measurements, where the measured roughness value in the 200–1000 nm range demonstrates that differences between them could not influence the results because the highly non-homogeneous topography of the sample could obscure the small differences due to organic/bio adhesion.

**Limitations:** However, there are limitations to our study, such as the low number of samples (only three CAM/CAD materials) and the non-variation in external factors (shock temperature, high acidity media, and the presence of enzymes that mimic the oral environment were not considered). Another limitation is the use of DMEM as a control medium. Since the short incubation period (48 h) was selected to research the impact of the first (incipient) stage of development of gingival cells on the surface of CAM/CAD materials, it does not reflect the long-term behavior of these materials under clinical conditions. Our samples were prepared by simply casting them in a cylindrical shaped plastic well. Their roughness is given by the roughness of the plastic and should not differ much for a given material. However, their casting roughness will differ from one material to another due to the differences in viscosity. A subsequent study is underway to assess the stability of the CAD/CAM materials following aggressive treatment in synthetic saliva to provide more clinically relevant insights into material stability. An important outline is that the evaluation of the cell influence should be extended to include the restoration’s margin since different materials have different margin adaptation. A recent paper [61] found that zirconia has the lowest marginal gap value (determined by scanning electron microscopy), followed by composite materials and, finally, by lithium disilicate. This affect biofilm formation as well as cell adaptation (e.g., the smaller the gap, the better the adaptation). A follow-up study will include margin adaptation as well as extension of the incubation period to 30 days/ 6 months.

## 5. Conclusions

The results of the various testing of the samples following cell culture and DMEM incubation show that materials are extremely stable and show little surface or structural modifications. This is supported by Raman spectroscopy, which revealed no structural modifications after the culture, and the SEM, AFM, and microhardness measurements, which indicated that the surface of the samples following biocorrosion is stable and not subjected to any visible modifications in terms of topography. The EDS results show that organic matter and biocorrosion do appear on the surface of the CAM/CAD materials and variate the elemental analysis (particularly for sample #2). The morphology and structure of the studied composites are stable under the investigated conditions. For those preliminary conditions, the investigated CAM/CAD materials are valid dental restoration products.

## Figures and Tables

**Table 1:** Compositions of the experimental materials involved in this study (according to manufacturer documentation); each sample is labeled by number.

Sample No	Commercial Name (Manufacturer)	Composition
#1	Trinia (SHOFU, Kyoto, Japan)	Glass fiber: 55 wt% + epoxy matrix resin: 45 wt%
#2	Coritec ZrO (IMES-ICORE GMBH—Eiterfeld, Germany)	ZrO_2_ + Y_2_O_3_ + HfO_2_ > 99 wt%, Al_2_O_3_ ˂ 0.5 wt%, other oxides 0.25 wt%
#3	Vita Enamic (Vita Zahnfabrik, Bad Sackingen, Germany),	UDMA, TEGDMA. Filler: Feldspar ceramic enriched with aluminum oxide, 86 wt%

Legend: UDMA: urethane dimethacrylate, TEGDMA: triethylene glycol dimethacrylate.

**Table 2:** The assignment of the Raman bands for the three investigated materials.

#1 (Trinia)	#2 (Coritec)	#3 (Vita)	Assignment	Ref.
	316 cm^−1^		Tetragonal ZrO_2_ (symmetry B_1g_)	[41]
		343 cm^−1^	C-C aliphatic chain	[42]
392 cm^−1^		383 cm^−1^	Si-O stretch in glass	[42]
	462 cm^−1^		Tetragonal ZrO_2_ (symmetry E_g_)	[41]
	608 cm^−1^		Cubic lattice of the ZrO_2_	[43]
639 cm^−1^	643 cm^−1^		Tetragonal ZrO_2_ (symmetry E_g_)—#2	[41]
668 cm^−1^			C-H deformation (-CH=CH-) cis	[44]
		706 cm^−1^	Mono-substituted C-H deformation out-of-plane	[44]
736 cm^−1^			CH_3_ aliphatic/-CH_2_- rocking of organic polymer *	[45]
810 cm^−1^			C-H deformation out-of-plane of the organic polymer	[44]
820 cm^−1^			C-H deformation out-of-plane of the organic polymer	[44]
914 cm^−1^			O-H deformation (aromatic carboxylic acids)	[44]
		967 cm^−1^	C-H deformation	[44]
1010 cm^−1^			C-O-C stretch in alkyl-aryl ethers	[44]
1113 cm^−1^		1118 cm^−1^	Si-O-CH_2_ stretch	[44]
1186 cm^−1^			P=O stretch/P-O-C strech	[44]
	1253 cm^−1^		P=O stretch	[44]
		1288 cm^−1^	Si-CH_3_ deformation	[44]
	1349 cm^−1^		S=O (anti-symmetrical)	[44]
1401 cm^−1^		1401 cm^−1^	Polynuclear aromatic polymers/C=O stretch (sym.)	[44]
	1428 cm^−1^		C-O stretch combined with O-H deformation in aromatic carboxylic acids	[44]
	1536 cm^−1^	1536 cm^−1^	In-plane ring deformation	[44]
1608 cm^−1^			C=C aromatic	[46]

* This band splits into two components in the solid state.

## Data Availability

Raw data can be made available freely upon request by the corresponding authors.

## Supplementary Materials

The following supporting information can be downloaded at: https://www.mdpi.com/article/10.3390/polym17020221/s1, Figure S1: AFM images for all samples; Table S1: EDS results for sample #1 for major and minor elements by weight% as an average over 3 measurements; Table S2: EDS results for sample #2 for major and minor elements by weight% as an average over 3 measurements; Table S3: EDS results for sample #1 for major and minor elements by weight% as an average over 3 measurements; Table S4: Full microhardness testing of the samples; Table S5: Rq values; Table S6: Ra values.
