# Supplementary material for: A Preliminary Stability Assessment of Three State-of-the-Art CAD/CAM Materials Under Human Gingival Cell Culture
Source: Polymers (Basel). 2025 Jan 17;17(2):221. doi: 10.3390/polym17020221 (PMC11769571; doi:10.3390/polym17020221)
Supplement: Supplementary file 1 [file polymers-17-00221-s001.zip › polymers-3382203-supplementary.pdf]

Stability assessment of three state-of-the-art CAD/CAM materials under human gingival cell culture

Gatin Eduard[1, 2*], Stefan Iordache[3], Ana Maria Iordache[3], Totan (Ripsvki) Alexandra[4], Antoniu Moldovan[5], Catalin Luculescu[5*]

[1] University of Medicine ''Carol Davila'', Faculty of Medicine, Bucharest, Romania; masterdent2009@yahoo.com (G.E.);
[2] University of Bucharest, Faculty of Physics, Atomistilor 405, Magurele, Ilfov, Romania;
[3] Optospintronics Department, National Institute for Research and Development in Optoelectronics—INOE 2000, Magurele, Romania; stefan.iordache@inoe.ro (S.I.); ana.iordache@inoe.ro (A.M.I.);
[4] University of Medicine "Carol Davila", Faculty of Dentistry, Bucharest, Romania; email Alexandra
[5] National Institute for Laser, Plasma and Radiation Physics, Magurele, Romania; crluculescu@yahoo.com (C.L.); antoniu.moldovan@inflpr.ro (A.M.)
* Correspondence: catalin.luculescu@inflpr.ro (C.L.); masterdent2009@yahoo.com (G.E.)

Table 1S. EDS results for sample #1 for major and minor elements by weight% as an average over 3 measurements

	C	O	Na	Al	Si
Raw	40.49	50.41	2.15	2.17	2.32
Medium	40.76	52.27	1.69	1.64	2.33
Culture	40.94	51.21	2.01	1.53	1.71

Table 2S. EDS results for sample #2 for major and minor elements by weight% as an average over 3 measurements

	C	O	Zr	Y
Raw	4.03	37.8	52.7	3.42
Medium	10.31	36.5	47.18	3.79
Culture	2.69	36.8	54.54	3.956

Table 3S. EDS results for sample #1 for major and minor elements by weight% as an average over 3 measurements

	C	O	Na	Al	Si	K
Raw	9.89	42.81	7.66	11.316	24.06	4.076
Medium	8.28	45.06	8	11.056	23.65	3.73
Culture	10.05	50.2	10.7	9.1	16.7	3.25

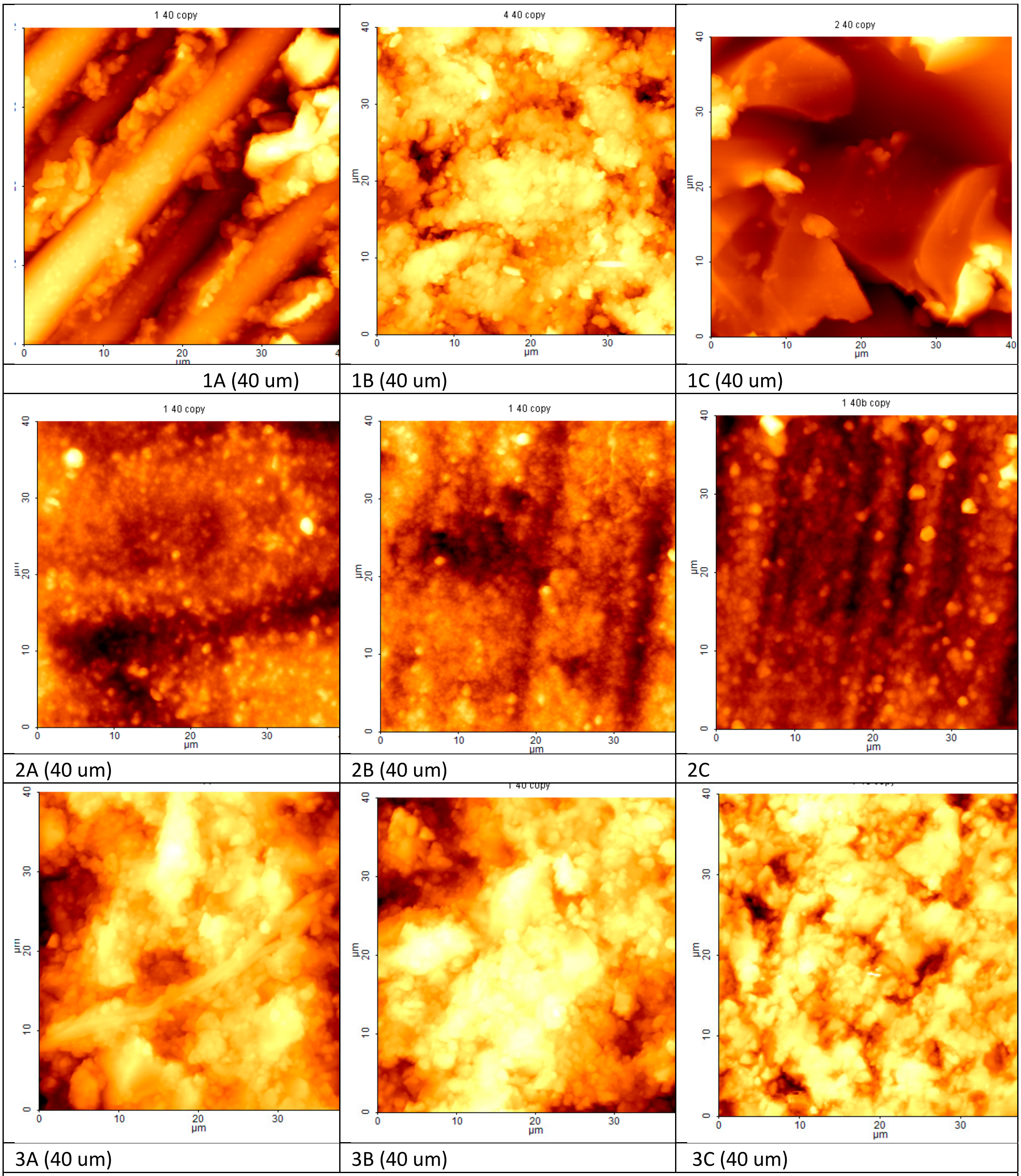

Figure 1S. AFM images for all samples

Table 4S. Full microhardness testing of the samples

Load	25gf	25gf	25gf	25gf	25gf	25gf	100gf	100gf	100gf	25gf	25gf	25gf	25gf
Sample	A_1	B_1	C_1	A_2	B_2	C_2	A_2	B_2	C_2	A_3glass	A_3	B_3	C_3
HV	22.5	11.9	11.2	604.1	372.2	670.5	1045.2	1002.6	984.4	91.6	225	204	193.7
	21.5	8.8	9.7	564.8	462.2	726.2	1082.2	1035.1	710.1	28.1	210.2	224.7	179.5
	18.9	6.5	10.6	516,4	454	574.9	1153.4	1124.8	615.7	36.1	234	240.5	219.1
Average	20.96	9.06	10.5	595.1	429.46	657.2	1093.6	1054.1	770.06	51.93	223.06	223.06	197.43
SD	1.85	2.70	0.75	74.60	49.76	76.52	54.99	63.29	191.52	34.58	12.01	18.30	20.06

Table 5S. Rq values

	Rq (nm)								
	1A	1B	1C	2A	2B	2C	3A	3B	3C
	922	676	657	256	268	302	1108	1026	264
	1039	314	903	287	280	537	768	296	161
		612						483	
Mean	980.5	534	780	271.5	274	419.5	938	389.5	212.5
SD	82.73149	193.1942	173.9483	21.92031	8.485281	166.1701	240.4163	132.229	72.832
RSD(%)	8.437684	36.17869	22.30106	8.073779	3.096818	39.61146	25.63074	33.94839	34.27388

Table 6S. Ra values

	Ra (nm)								
	1A	1B	1C	2A	2B	2C	3A	3B	3C
	753	527	514	198	214	226	900	830	207
	759	248	688	215	229	439	616	236	134
		485						380	
Mean	756	420	601	206.5	221.5	332.5	758	308	170.5
SD	4.242641	150.4294	123.0366	12.02082	10.6066	150.6137	200.8183	101.8234	51.6188
RSD(%)	0.561196	35.81652	20.47198	5.821218	4.788534	45.29737	26.49318	33.05954	30.27495
